# Myocardial Flow Reserve Measurement During CZT-SPECT Perfusion Imaging for Coronary Artery Disease Screening: Correlation With Clinical Findings and Invasive Coronary Angiography—The CFR-OR Study

**DOI:** 10.3389/fmed.2021.691893

**Published:** 2021-06-04

**Authors:** Matthieu Bailly, Frédérique Thibault, Maxime Courtehoux, Gilles Metrard, Denis Angoulvant, Maria Joao Ribeiro

**Affiliations:** ^1^Nuclear Medicine Department, CHR ORLEANS, Orleans, France; ^2^Nuclear Medicine Department, CHRU TOURS, Tours, France; ^3^Cardiology Department, CHRU TOURS, Tours, France; ^4^EA4245 T2i, Tours University, Tours, France; ^5^UMR 1253, iBrain, Université de Tours, Inserm, Tours, France

**Keywords:** myocardial blood flow, myocardial flow reserve, CZT-SPECT, cardiovascular risk, invasive coronary angiography

## Abstract

**Purpose:** The aim of this study was to assess the results of cadmium zinc telluride (CZT)- single-photon emission computed tomography (SPECT) myocardial flow reserve (MFR) in coronary artery disease (CAD) screening regarding clinical risk and its correlation to invasive coronary angiography (ICA).

**Methods:** A total of 137 patients (61 male and 76 female) referred for CAD screening myocardial perfusion imaging (MPI) between November 2018 and April 2020 were included in the CFR-OR prospective trial. The 10-year risk of cardiovascular death according to the European Society of Cardiology (SCORE) was calculated. SPECT 1-day 99mTc-tetrofosmin protocol was acquired on CZT cardiac-dedicated pinhole cameras. Low-dose thoracic CT was used for coronary calcium score (CCS) evaluation. ICA, when performed within 3 months, was also analyzed.

**Results:** Mean SCORE and mean global MFR were, respectively, 4 ± 3.1% and 2.50 ± 0.74; 34 patients had impaired CFR (using a threshold of 2). There was a significant inverse correlation between MFR and SCORE (*p* = 0.006), gender (*p* = 0.019), and number of cardiovascular risk factors (*p* = 0.01). MFR was significantly reduced in patients with CCS above 1 (*p* = 0.01). No significant correlation was found between MFR and individual cardiovascular risk factors (dyslipidemia, hypertension, diabetes, or family history of CAD). A total of 23 patients underwent ICA. Global MFR SPECT sensitivity and specificity were 83.3 and 100 %, respectively, with an area under the curve of 0.94.

**Conclusion:** Adding MFR to SPECT MPI for CAD screening on CZT camera may contribute to high-risk patient identification and enhance diagnostic performances. MFR could help physician decision to perform ICA.

## Introduction

Stress single-photon emission computed tomography (SPECT) myocardial perfusion imaging (MPI) is commonly used for diagnosis and risk stratification of patients with suspected coronary artery disease (CAD) (1). Conventional SPECT MPI, using visual analysis and semi-quantitative parameters, assesses the presence, extent, and degree of myocardial ischemia and/or necrosis.

Myocardial blood flow (MBF) and myocardial flow reserve (MFR), calculated using Positron emission tomography (PET) MPI provide added diagnostic (2, 3) and prognostic (4) information than relative perfusion analysis alone. Cardiac PET is not as widely available as SPECT. The edge of cadmium zinc telluride (CZT) detectors in SPECT allow time–activity curve (TAC) acquisitions without any rotation, with higher sensitivity and temporal resolution (5). Some studies with experimental animal models demonstrated the capability to estimate absolute MBF and MFR estimation by SPECT (6). Several studies have shown that the clinical measurement of MBF and MFR using dynamic CZT-SPECT MPI with ^99m^Tc radiopharmaceuticals is technically possible, resulting in similar MFR when compared to PET (7–10). One potential limit is the unfavorable properties of the currently available flow SPECT tracers, hampering accurate MBF quantification due to non-linear extraction fraction with roll-off at higher flow values.

CZT SPECT MPI quantitative flow parameters in the diagnosis of CAD have not been extensively reported until now. One recent study showed that MBF and MFR provided diagnostic information for patients with suspected or known CAD, in addition to the conventional analysis of qualitative and semi-quantitative parameters (11). We recently reported a case of MPI for CAD screening with normal relative MPI, high calcium score, and strongly impaired MBF and MFR. In this patient, coronary angiography confirmed extended CAD with three-vessel disease (12).

In this study, we prospectively evaluated the clinical correlations of SPECT MFR in patients referred for CAD screening and its correlation to invasive coronary angiography (ICA).

## Materials and Methods

### Patient Population

From October 2018 to June 2020, 137 patients without known CAD referred for SPECT MPI with MBF and MFR quantification and addressed to two Nuclear Medicine departments were prospectively enrolled in the CFR-OR trial (clinicaltrials.gov unique identifier NCT03586492). Every patient received information and gave informed consent. The study protocol was approved by the local and regional ethics committees (CPP Ouest III), and the procedures were in accordance with the Declaration of Helsinki. The inclusion criterion was CAD screening MPI.

The exclusion criteria included previously known CAD, absolute common contraindication to vasodilators (severe hypotension, second- or third-grade atrioventricular block, and recent myocardial infarction), previous cardiac surgery, pregnancy, or active breastfeeding.

**Figure 1:**
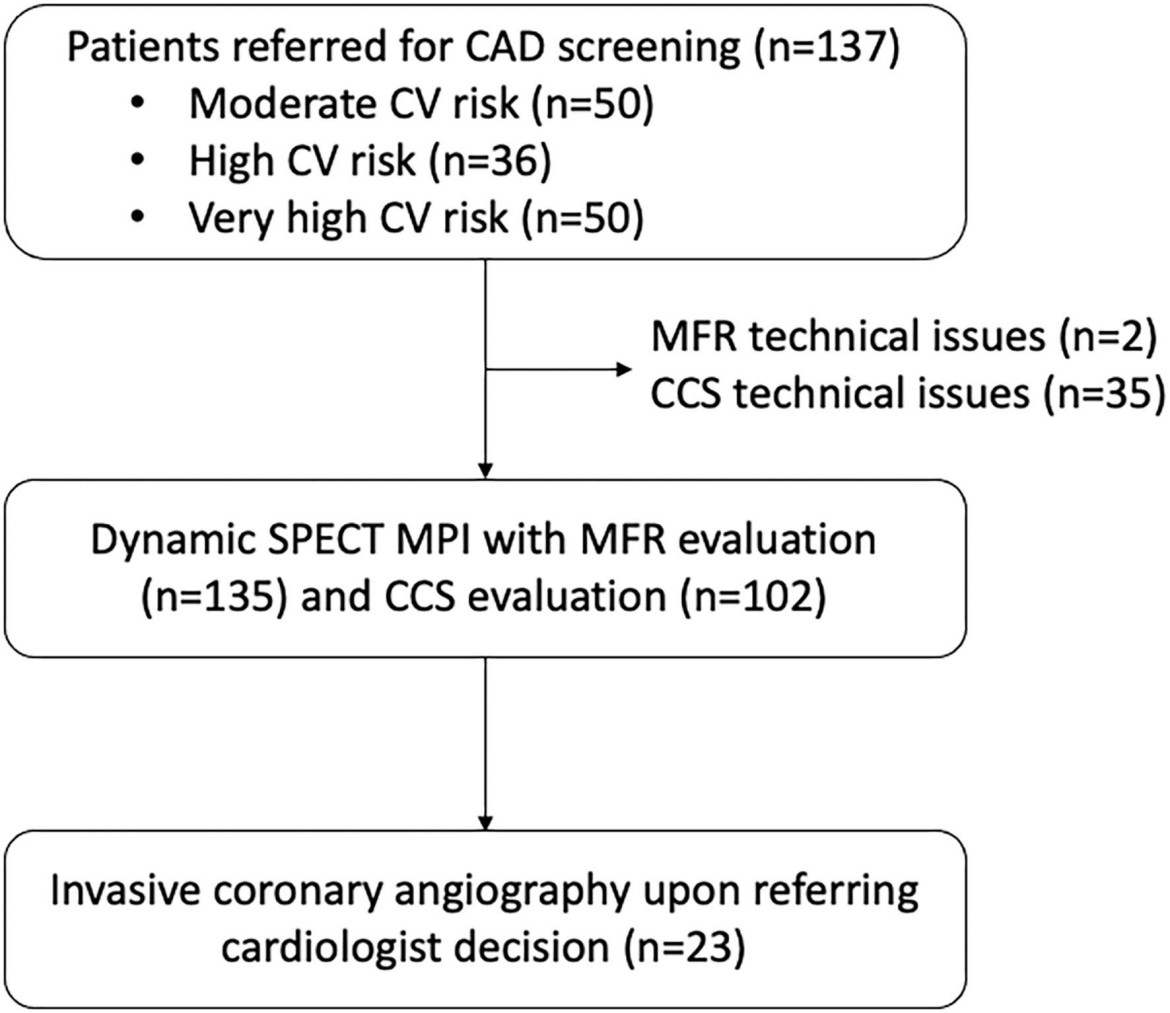
Flow chart of the study.

Technical issues were reported for MBF and MFR measurement in two patients (late acquisition after injection) and for CCS evaluation in 35 patients (movement artifacts, CT breakdown). A flow chart of the study is displayed in Figure 1.

### Estimation of 10-Year Risk of Fatal Cardiovascular Events

A patient's individual estimation of 10-year risk of cardiovascular mortality was assessed using the ESC low-risk regions of Europe SCORE based on age, gender, smoking, systolic blood pressure, and total blood cholesterol concentration (13). Accordingly, very-high-risk patients were defined as having ≥10% risk, with high-risk patients having 5–9% and moderate-risk patients having 3–4%.

### SPECT Acquisition

**Figure 2:**
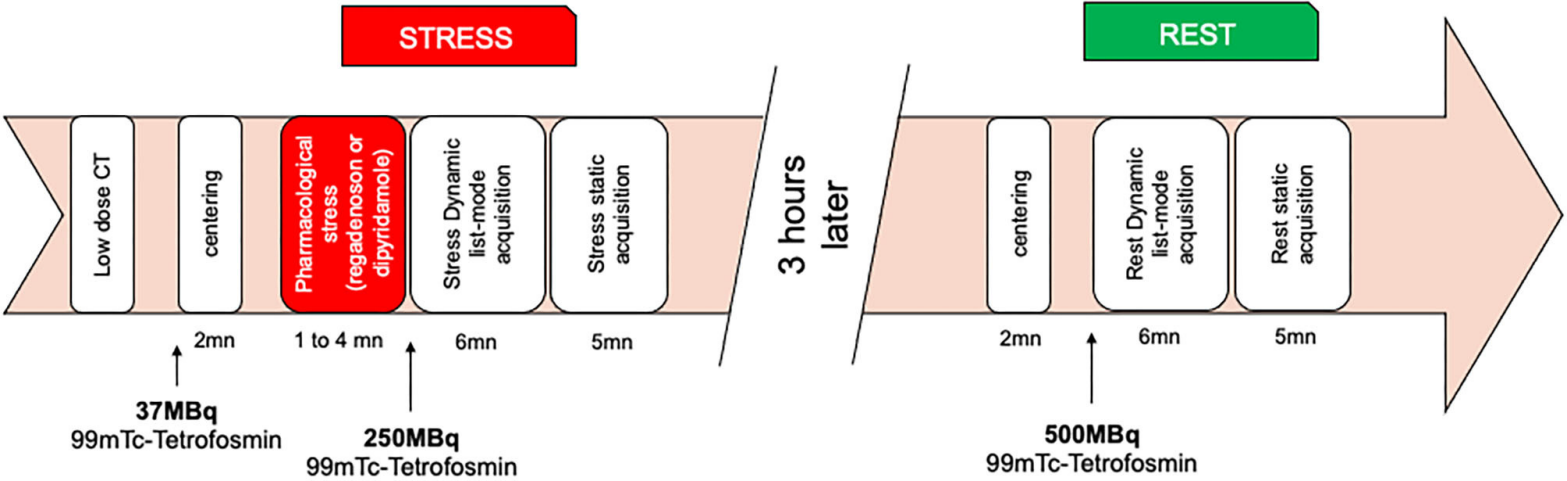
Dynamic cardiac myocardial perfusion imaging protocol.

List-mode acquisitions were performed on two same Discovery NM530c cardiac CZT cameras (General Electric Healthcare, Haifa, Israel) in both departments. An initial injection of 37 MBq of ^99m^ Tc-tetrofosmin was used to center the patient's heart in the field of view. Pharmacological stress was then performed using either a regadenoson (400 μg) injection or a dipyridamole perfusion (0.56 mg/kg), immediately followed with 250 MBq of ^99m^Tc-tetrofosmin bolus injection at hyperemia peak and then flushed by 50 ml of saline to ensure the consistent delivery of a tight bolus. Rest dynamic acquisition was realized 3 h later, with a similar injection of 500 MBq of ^99m^Tc-tetrofosmin, in agreement with the recent work of Zoccarato et al. (14). Non-ECG-gated, free-breathing low-dose CT was acquired on a conventional hybrid gamma-camera [Discovery NM670pro hybrid gamma-camera: 120 kV/20–120 mA, pitch 1.375:1, slice 1.25 mm (General Electric Healthcare, Haifa, Israel) or Symbia T2: 130 kV/30 mA, slice 2.5 mm (Siemens Healthineers, Erlangen, Germany)], placing the patient in the same position as on the CZT gamma-camera, with the arms above the head. Those parameters were in agreement with the recent best practices for CT-based attenuation correction (AC) and coronary calcium score (CCS) in nuclear cardiology (15). The procedure timeline is displayed in Figure 2. “Static” acquisitions could consist of supine and/or prone imaging for visual MPI analysis. Visual MPI analysis was performed by nuclear medicine board-certified physicians and checked by the principal investigator. Negative MPI was defined as no perfusion defect and no indirect sign of three-vessel disease (diminution of ejection fraction at stress, transient ischemic dilatation, and right ventricle visualization). No semi-quantitative analysis was performed, but defects (ischemia and/or necrosis when found) were quantified using the 17-segment model.

### SPECT MBF and MFR Quantification

Dynamic SPECT was reconstructed using Corridor 4DM(GE) software, v2015.0.2.66 (INVIA, Ann Arbor, MI, USA), on a Xeleris 4DR workstation (General Electric Healthcare, Haifa, Israel). SPECT initial list-mode was resampled into 12 × 10- and 8 × 30-s frames. Endocardial and epicardial left ventricle (LV) surfaces were algorithmically estimated from summed myocardial images after 2-min acquisition time. LV myocardial tissue TACs were nearest-neighbor-sampled at the center of each 460 polar map sectors at the midwall surface, on all time frames. Global and regional [left anterior descending artery (LAD), left circumflex (LCx), right coronary artery (RCA)] TACs were automatically generated by the software. A 3D box region of interest within the LV/left atrium (LA) blood pool was used for MBF estimation, placed on the valve plane, and centered across all time frames by the same physician. This region of interest was designed to sample both the LV and LA cavities (two pixels in width in the short axis and 30 mm in length in the long axis) (16). Retention rate *R* was calculated using the net retention model of Leppo and Meerdink (17) and Yoshida et al. (18) according to the following equation:

$$R=\text{MBF}\times E=\frac{\frac{1}{PV\times (t3-t2)}\int_{\text{t2}}^{\text{t3}}\text{P}(t)-\text{Sm}\times \text{Ca}(t)\text{dt}}{\left(\text{CF}\right)\int_{\text{0}}^{\text{t1}}\text{Ca}(t)-\text{Sb}\times \text{P}(t)\text{dt}}$$

MBF, *E*, and P(t) were, respectively, the myocardial blood flow, the extraction fraction, and the total myocardial tracer concentration or tissue TAC. Ca(t) represents the arterial concentration of the tracer or blood TAC and PV the partial volume value (set to 0.6). We set the correction factor for myocardial density (CF) to 1. Sm and Sb represented, respectively, the spillover from the blood pool activity to the myocardium estimated from compartmental analysis (Sm set to 0.4) and the spillover from the myocardium to the blood pool activity (assuming this one was negligible, it was set to 0). Integration limits t1, t2, and t3 corresponded to the end of the blood pool phase at 1 min for t1 and to the average tissue activity, from 1 to 2 min for t2 and t3. Those limits were adjusted to the peak of the blood TAC. The uptake rate K1 was related to MBF using the following Renkin–Crone equation according to Leppo (17), where *A* = 0.874 and *B* = 0.443:

$$\begin{array}{l} \text{K}1=\text{MBF }*\;\left(1-{\text{A }*\text{ e}}^{-\frac{B}{MBF}}\right) \end{array}$$

Because our previous results (19) showed no difference in terms of MFR whether attenuation correction was applied or not, we did not apply it in this study. All MBF and MFR values are presented without attenuation correction.

### Coronary Calcium Score Evaluation

CCS was estimated on the low-dose CT as the sum of CCS in the three main coronary arteries according to the method described by Agatston et al. (20). This evaluation was made visually. Horos software was used only for training and to support the visual estimation (free and open-source code software program that is distributed free of charge under the LGPL license at Horosproject.org and sponsored by Nimble Co LLC d/b/a Purview in Annapolis, MD USA). Previous studies demonstrated a high agreement between CCS evaluated on attenuation correction CT for SPECT with the Agatston classical CCS and excellent inter-reader reproducibility (21, 22). The authors recommended that the degree of atherosclerosis should be assessed by means of estimating CCS on the CT for attenuation correction. In this study, the evaluation was performed by the same physician, and CCS score was classified in two categories: 0 CCS score and CCS ≥1.

### Invasive Coronary Angiography

ICA was performed upon the referring cardiologist's decision, after the MPI, MBF, and MFR results, according to clinical practice (the fractional flow reserve measurements were left at the angiographer's decision). Coronary angiograms were visually assessed by the experienced interventional cardiologist responsible for the procedure. The angiograms were assessed according to the clinical routine, taking into account available clinical data and patient history. According to the recent ESC guidelines defining very-high-risk patients in need of secondary prevention intervention, we considered all patients with significant coronary artery plaque according to the angiographer's conclusion, i.e., ≥50% narrowing of the diameter of the lumen of the arteries (13).

### Statistical Analysis

Continuous variables are presented as means ± standard deviation. Categorical variables are provided as total numbers in percent. Gaussian distribution was assessed using the D'Agostino–Pearson normality test. When analyzing differences between the two groups, we applied independent-sample *t*-test when comparing continuous variables (SCORE, MFR) and the χ^2^ test or Fisher's exact test as appropriate when comparing categorical variables (CCS). ANOVA test was performed to compare multiple, not paired, groups with Gaussian distribution (clinical risk, number of risk factors, and CCS). The non-normal distributed variables were analyzed using the Mann–Whitney *U* test (some small groups regarding micro-albuminuria status or risk factors) and the Kruskal-Wallis test for multiple-group comparisons (MBF and MFR values in three CV risk groups). Pearson's correlation coefficients were computed between variables. Linear regression was used to analyze correlations between MFR, CCS, and other covariates (SCORE). Receiver operating characteristic (ROC) curves were calculated for MFR and stress MBF according to ICA results. *p* < 0.05 was considered statistically significant. All analyses were performed using Prism 9.

## Results

### MFR and CCS According to Clinical Findings

A total of 137 patients (61 male and 76 female) were included and classified in the three SCORE-estimated 10-year CV mortality risk groups: 50 moderate, 37 high, and 50 very high (Table 1).

**Table 1:** Patients' demographics.

**Number of patients**	**137**
Gender Male/female	61 (45%)/76 (55%)
Mean age ± SD (years)	68 ± 9.3 (41–87)
BMI ± SD (kg/m^2^)	28.3 ± 5.4 (15-44)
Cardiovascular risk (CVR) factors	
Diabetes	49 (36%)
Hypertension	93 (68%)
Smoking	66 (48%)
Dyslipidemia	88 (64%)
Family history of coronary artery disease	18 (13%)
Mean number of CVR factors	2.3 ± 1 (0–5)
Mean SCORE ± SD	4 ± 3.1 (1–17)
Score	
Moderate	50 (36.5%)
High	37 (27%)
Very high	50 (36.5%)
Mean MFR ± SD	2.5 ± 0.74 (0.81–4.8)
Mean stress MBF ± SD (ml/min/g)	1.50 ± 0.54 (0.55–3.8)
Mean rest MBF ± SD (ml/min/g)	0.65 ± 0.28 (0.17–1.7)
CCS (Agatston units) (*n* = 102)	
0	20 (19%)
≥1	82 (81%)

*SD, standard deviation; CVR, cardiovascular risk; SCORE, 10-year risk of cardiovascular death according to the European Society of Cardiology; MFR, myocardial flow reserve; MBF, myocardial blood flow; CCS, coronary calcium score*.

Mean SCORE and mean global MFR were, respectively, 4 ± 3.1% and 2.50 ± 0.74. The mean global stress MBF was 1.50 ± 0.54 ml/min/g. There were 35 patients who had impaired CFR (using a threshold of two), but only six of these patients also had impaired visual MPI.

**Figure 3:**
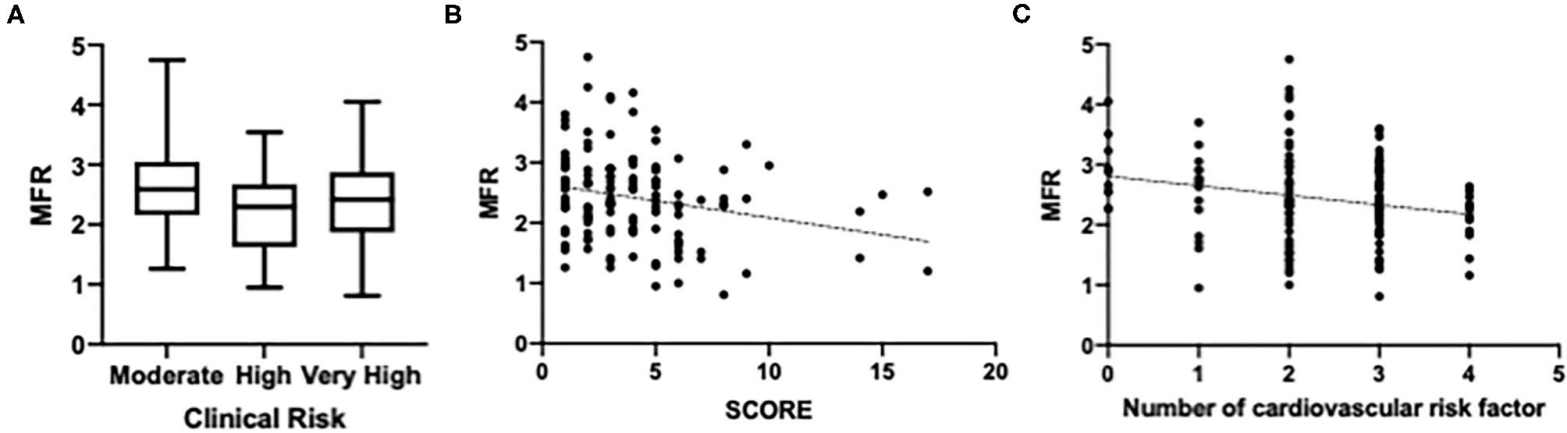
Myocardial flow reserve (MFR) according to clinical risk. **(A)** MFR according to CV mortality risk groups. **(B)** MFR correlated to SCORE-estimated 10-year cardiovascular (CV) mortality. **(C)** MFR correlated to the number of CV risk factors.

MFR was significantly different between the three CV mortality risk groups (*p* = 0.03) (Figure 3), being significantly reduced in high- and very-high-risk patients. CCS was not different according to risk categories (*p* = 0.32) (Table 2).

**Table 2:** Clinical findings, myocardial flow reserve (MFR), and coronary calcium score (CCS) according to cardiovascular risk (CVR) groups.

**CVR group**	**Moderate (*n* = 50)**	**High (*n* = 37)**	**Very high (*n* = 50)**	***p***
Gender male/female	21 (42%)/29 (58%)	27 (73%)/10 (27%)	20 (40%)/30 (60%)	0.09
Mean age ± SD (years)	66 ± 10 (41–87)	69 ± 7.9 (52–85)	69 ± 9.3 (44–87)	0.03
BMI ± SD (kg/m^2^)	27 ± 5.3 (18–40)	28 ± 5.7 (18–41)	29 ± 5.3 (15–44)	0.17
CVR factors				
Diabetes	1 (2%)	2 (5%)	46 (92%)	<0.001
Hypertension	30 (60%)	35 (70%)	38 (76%)	0.14
Smoking	25 (50%)	31 (62%)	21 (42%)	0.008
Dyslipidemia	23 (46%)	29 (58%)	41 (82%)	0.003
Family history of coronary artery disease	12 (24%)	8 (16%)	4 (8%)	0.01
Mean number of CVR factors	1.8 ± 1.1 (0–4)	2.1 ± 1 (0–4)	2.9 ± 0.65 (2–5)	<0.001
Mean SCORE ± SD	2.4 ± 1.1 (1–10)	4.9 ± 2.7 (1–15)	4.3 ± 4 (2–17)	0.008
Mean MFR ± SD	2.6 ± 0.74 (1.3–4.8)	2.4 ± 0.77 (0.95–4.6)	2.4 ± 0.74 (0.81–4.1)	0.03
Mean stress MBF ± SD (ml/min/g)	1.50 ± 0.61 (0.6–3.8)	1.50 ± 0.57 (0.55–3.7)	1.50 ± 0.49 (0.66–2.9)	0.06
Mean rest MBF ± SD (ml/min/g)	0.59 ± 0.23 (0.17–1.4)	0.68 ± 0.30 (0.17–1.5)	0.66 ± 0.29 (0.31–1.7)	0.48
CCS (Agatston units)				0.32
0	10 (20%)	6 (16%)	4 (8%)	
1–100	14 (36%)	13 (26%)	14 (28%)	
100–400	8 (16%)	8 (16%)	8 (16%)	
≥401	2 (4%)	5 (10%)	10 (20%)	

**Figure 4:**
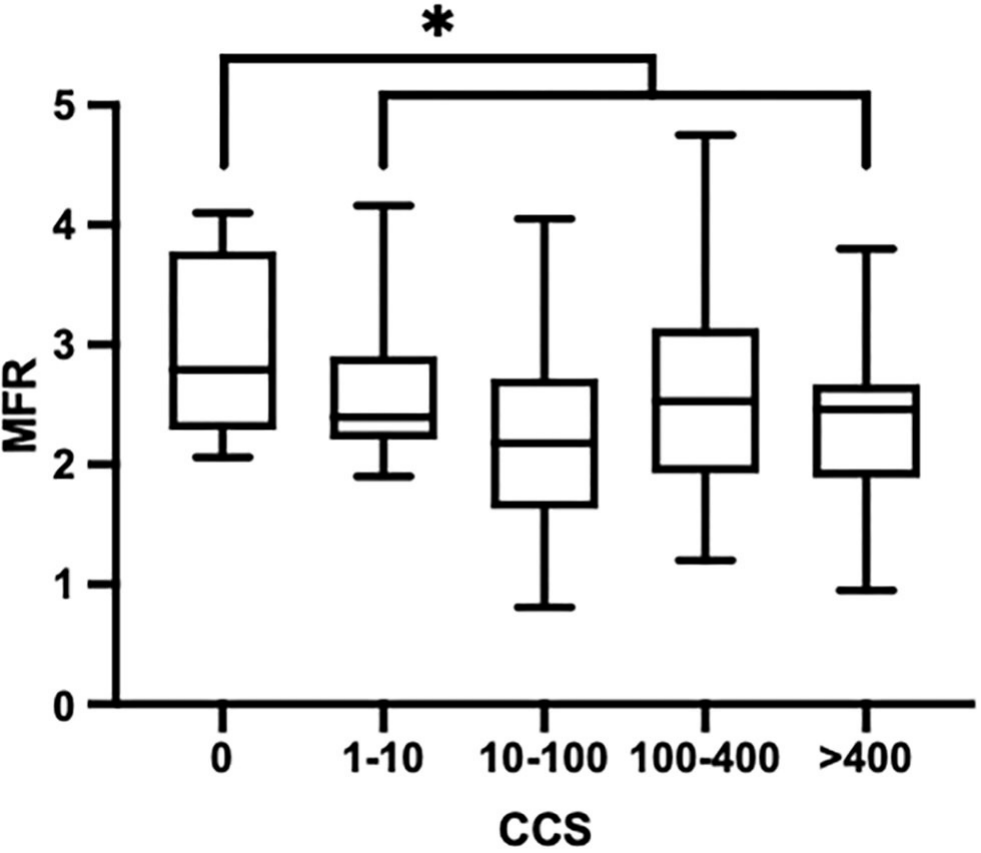
Myocardial flow reserve according to coronary calcium score. *p < 0.05.

There was a significant inverse correlation between MFR and SCORE (*r* = −0.24; *p* = 0.006), gender (*r* = −0.20; *p* = 0.019), and number of cardiovascular risk factors (*r* = −0.22; *p* = 0.01). A significant correlation was also found between CCS and SCORE (*r* = 0.25; *p* = 0.013) and gender (*r* = 0.22; *p* = 0.027). MFR was significantly reduced in patients with CCS ≥1 (*p* = 0.002) (Figure 4), with a mean global LV. MFR was 2.8 ± 0.67 and 2.3 ± 0.76, respectively, for patients with 0 CCS score and CCS score ≥1.

Regarding CV risk factors, CCS was significantly higher in smokers (*p* = 0.05), but we did not find other significant correlations between MFR or CCS and individual risk factors (dyslipidemia, hypertension, diabetes, or family history of coronary artery disease; *p* of at least 0.18) (Table 3). We noticed a significant inverse correlation between smoking and stress MBF (*p* = 0.02).

**Table 3:** Correlation between cardiovascular risk factors, MFR, stress MBF, and CCS.

**Cardiovascular risk factor**	**Correlation with global MFR (*p*)**	**Correlation with global stress MBF (*p*)**	**Correlation with global CCS (*p*)**
Diabetes	*r* = −0.081	*r* = −0.035	*r* = −0.018
	*p* = 0.608	*p* = 0.692	*p* = 0.857
Hypertension	*r* = 0.028	*r* = 0.076	*r* = 0.134
	*p* = 0.475	*p* = 0.383	*p* = 0.187
Dyslipidemia	*r* = −0.028	*r* = −0.032	*r* = −0.024
	*p* = 0.608	*p* = 0.719	*p* = 0.816
Smoking (active or <3 years)	*r* = −0.006	*r* = −0.109	*r* = −0.014
	*p* = 0.706	*p* = 0.022	*p* = 0.893
Family history of coronary artery disease	*r* = 0.006	*r* = −0.060	*r* = −0.091
	*p* = 0.764	*p* = 0.491	*p* = 0.373
Number of cardiovascular risk factors	*r* = −0.222	*r* = −0.048	*r* = 0.087
	*p* = 0.010	*p* = 0.577	*p* = 0.388
SCORE	*r* = −0.217	*r* = −0.097	*r* = −0.253
	*p* = 0.006	*p* = 0.276	*p* = 0.013

*MFR, myocardial flow reserve; MBF, myocardial blood flow; CCS, coronary calcium score; SCORE, 10-year risk of cardiovascular death according to the European Society of Cardiology*.

There were 77 patients who reported cardiovascular symptoms [chest pain (typical or atypical) or dyspnea]. MFR was not significantly different according to symptoms (*p* = 0.25).

Finally, MFR tend to be reduced in patients with high microalbuminuria (Table 4). Mean global MFR was 2.4 ± 0.76 and 2.2 ± 0.78 when albuminuria/creatinuria ratio was, respectively, ≤20 and >20 mg/g (all of these patients considered with high microalbuminuria were diabetic and/or hypertensive patients).

**Table 4:** MFR and MBF according to microalbuminuria.

	**No microalbuminuria <20 mg/g**	**Microalbuminuria >20 mg/g**	***p***
	**(*n* = 61)**	**(*n* = 26)**	
Diabetes	19 (31%)	14 (54%)	0.09
Hypertension	40 (66%)	20 (77%)	0.60
Mean stress MBF ± SD (ml/min/g)	1.5 ± 0.56 (0.55–3.8)	1.5 ± 0.72 (0.66–3.8)	0.62
Mean MFR ± SD	2.4 ± 0.76 (0.95–4.8)	2.2 ± 0.78 (0.81–3.8)	0.33

*MFR, myocardial flow reserve; MBF, myocardial blood flow; SD, standard deviation*.

### Coronary Angiography Findings

**Figure 5:**
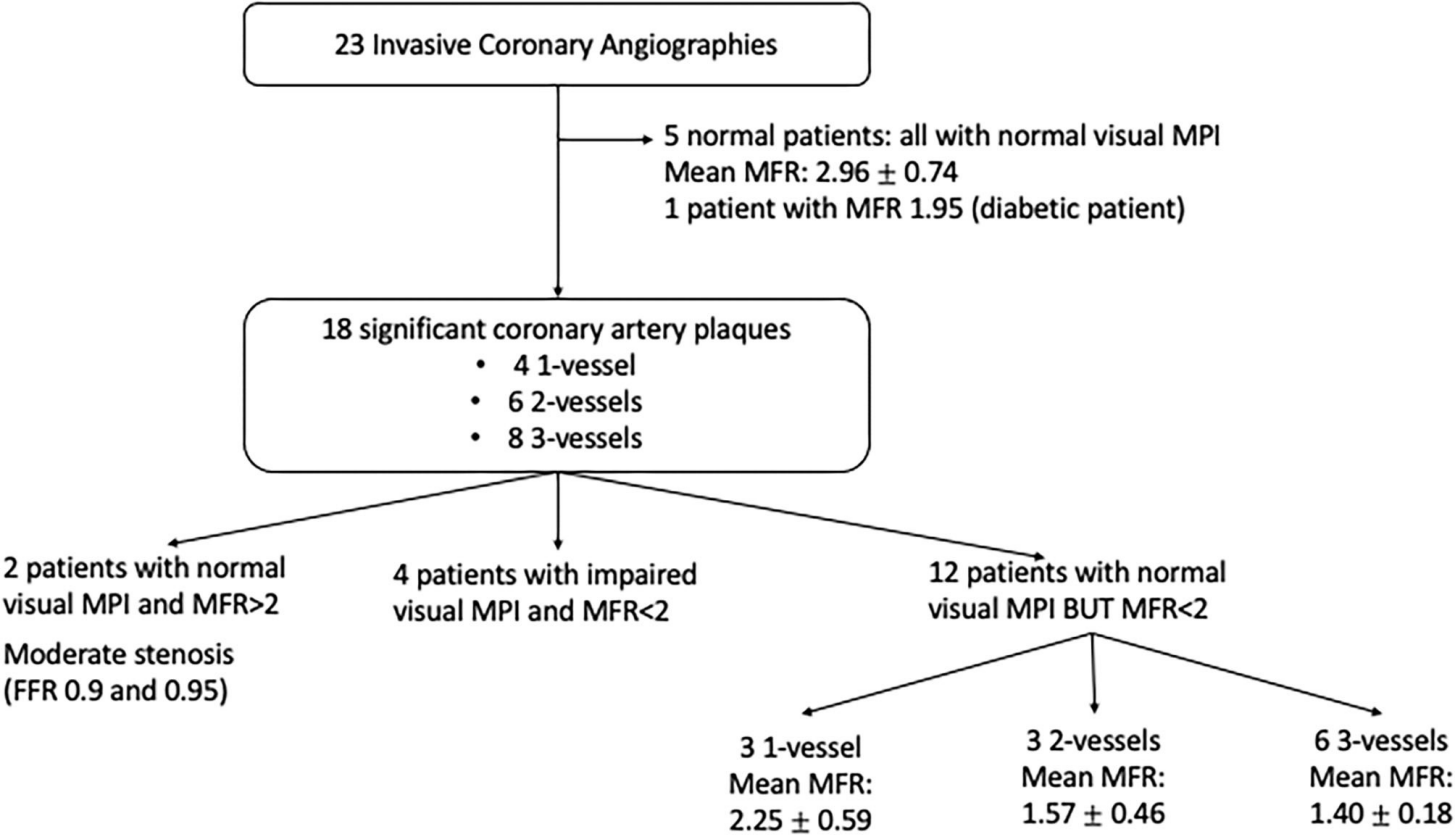
Myocardial perfusion imaging and myocardial flow reserve according to invasive coronary angiography results.

A total of 23 ICA were performed: five were considered normal, 18 showed significant coronary artery plaques qualifying for high-risk patients (four one-vessel, six two-vessel, and eight three-vessel disease). All normal ICA had normal visual MPI, and only one patient had a global MFR <2 (diabetic and symptomatic patient). Considering the 18 patients with significant coronary artery plaques, 16 had impaired MFR (two patients had global and regional MFR >2, but their stenosis was moderate, with negative FFR: 0.9 and 0.95), whereas only four patients had impaired visual MPI (Figure 5). CCS was significantly higher in patients with CAD: 90 ± 150 and 480 ± 660, respectively, for patients without CAD and with CAD (*p* = 0.02). The mean global MFR was also significantly lower in patients with CAD; stress MBF and MFR were both significantly lower in vessel territories with CAD (Table 5).

**Table 5:** MFR and MBF findings in patients with and without obstructive coronary artery disease (CAD).

	**Global-based analysis**			**Vessel-based analysis**		
	**Without CAD**	**With CAD**	***p***	**Without CAD**	**With CAD**	***p***
	**(*n* = 5)**	**(*n* = 18)**		**(*n* = 37)**	**(*n* = 32)**	
Mean stress MBF ± SD (ml/min/g)	1.6 ± 0.48 (1.3–2.4)	1.2 ± 0.54 (0.55–2.5)	0.13	1.5 ± 0.55 (0.61–2.6)	1.2 ± 0.63 (0.38–3)	0.009
Mean MFR ± SD	3 ± 0.74 (2.1–3.8)	1.7 ± 0.54 (0.81–2.9)	0.001	2.3 ± 1 (0.9–4.7)	1.6 ± 0.58 (0.49–3.3)	0.0007

*MFR, myocardial flow reserve; MBF, myocardial blood flow; SD, standard deviation*.

**Figure 6:**
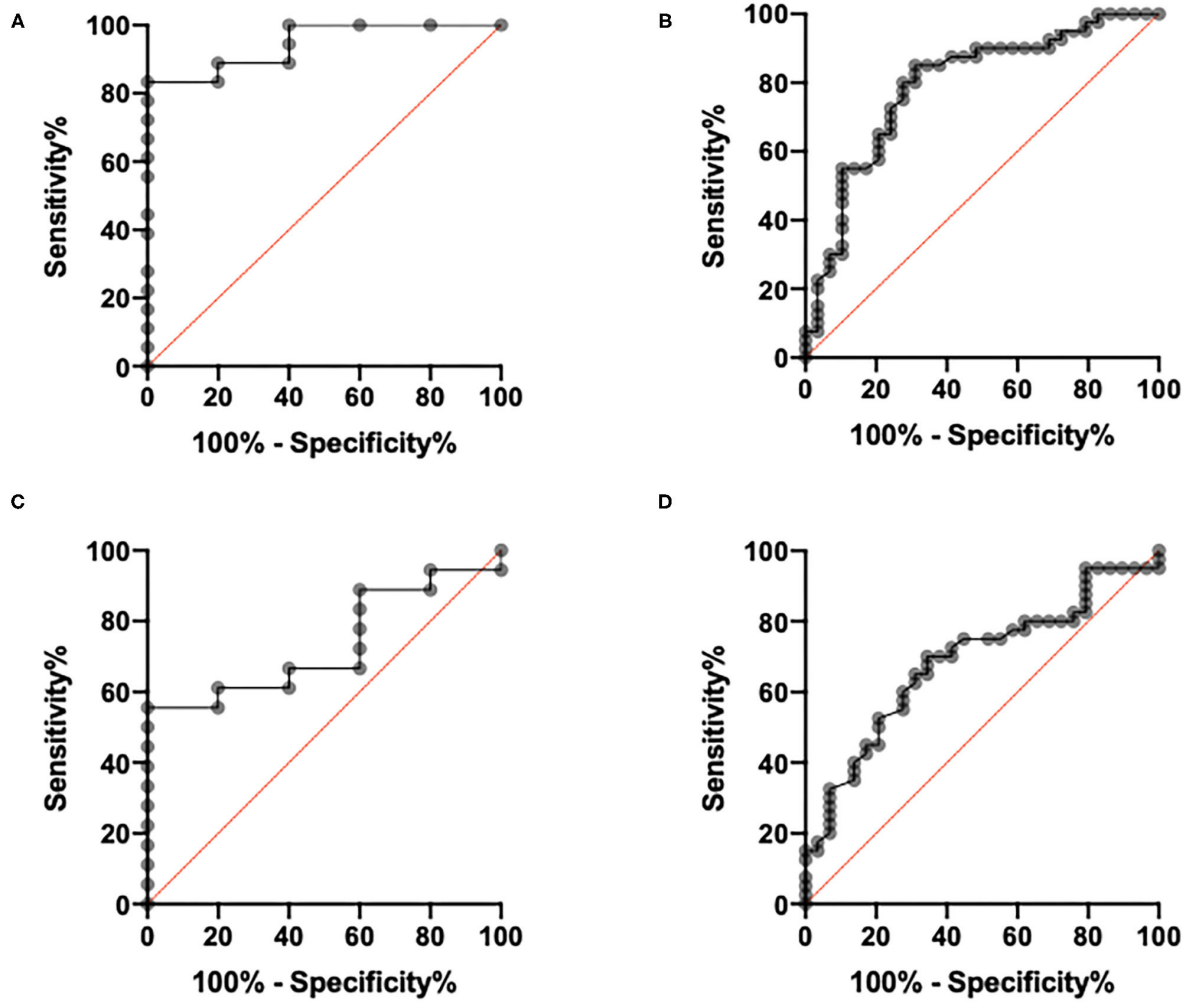
Receiver operating characteristic curves per vessel analysis and per patient analysis. **(A)** Per patient analysis, based on global myocardial flow reserve (MFR). **(B)** Per vessel analysis, based on regional MFR. **(C)** Per patient analysis, based on global stress myocardial blood flow (MBF). **(D)** Per vessel analysis, based on regional stress MBF.

Based on global MFR and with a threshold of 2, sensitivity and specificity were, respectively, 83.3 and 100%, and area under the curve (AUC) was 0.94 (Figure 6). Regarding regional MFR for the per-vessel analysis, using the same threshold of 2, sensitivity and specificity were, respectively, 85 and 62%, and AUC was 0.79. Based on the ROC curve, the best MFR threshold appeared to be 2.28 on a per-patient analysis, resulting in the best sensitivity and specificity compromise of 88.9% (IC 95%: 67.20–98.03%) and 80% (IC 95%: 37.55–98.87%), with a likelihood ratio of 4.44. The best threshold for regional MFR was 1.92, and this resulted in sensitivity and specificity of 84.4% (IC 95%: 68.25–93.14%) and 56.76% (IC 95%: 40.91–71.33%), with a likelihood ratio of 1.95. Based on global stress MBF, the best threshold appeared to be 1.28, resulting in the best sensitivity and specificity compromise of 61.1% (IC 95%: 38.62–79.69%) and 80% (IC 95%: 37.55–98.87%), with a likelihood ratio of 3.1. The same threshold for regional stress MBF resulted in sensitivity and specificity of 70% (IC 95%: 54.57–81.93%) and 65.52% (IC 95%: 47.35–80.06%), with a likelihood ratio of 2.03.

## Discussion

In this study, we found a significant reduction of global SPECT MFR in patients with high SCORE-estimated 10-year CV mortality risk, high number of CV risk factors, and CCS ≥ 1. In addition, our data suggest that global SPECT MFR may be a good predictor of angiographically significant coronary artery lesions.

Our SPECT results are consistent with the reported PET findings. MFR above 2 as assessed by PET has been recognized as a normal value, resulting in a very low rate of cardiac events (4, 23). With an excellent negative predictive value of nearly 1.0, independently of semi-quantitative perfusion results, it could safely exclude patients at high CV risk (24). However, despite of other risk factors, patients with MFR below 1.5 were shown to have a significantly worse prognosis (25). Our findings are also consistent with the recent SPECT studies from Acampa et al. the authors demonstrated a relationship between MPI findings and both hyperemic MBF and MFR obtained by CZT-SPECT, yet global MFR resulted as an independent predictor of CAD, and regional MFR was useful for the identification of obstructive CAD in the corresponding coronary artery. Their best thresholds were 2.6 for global MFR and 2.1 for regional MFR (26).

Although MFR appears to be a strong predictor of cardiovascular mortality, better than stress MBF, it is dependent on both hyperemic and rest flows. For example, low stress MBF with low rest MBF, in case of hibernating myocardium, could result in normal MFR; abnormal MFR could be found with normal stress MBF and high rest MBF. Thus, the integrated assessment of stress MBF and MFR helps to improve diagnostic performances (27, 28). According to our results, the best threshold seemed to be 1.28, but this finding should be put into perspective, knowing the great variability in the described flow values. Our MFR values are similar to other SPECT studies. Giubbini et al., Acampa, and Agostini et al. reported mean global MFR values of 2.18 ± 0.83, 2.44 ± 0.7, 2.60 ± 0.8, and 2.84 ± 0.81, respectively (8–10, 26). Regarding stress MBF values, results from previous works are quite heterogeneous. Our stress MBF values are consistent with those reported by Fang et al., with mean stress MBF of 1.77 ± 0.46 (29), but some authors reported higher values, with mean stress MBF of 3.18 ± 0.95, 2.40 ± 0.7, 3.27 ± 1.1, and 2.3 ± 0.97, respectively, for Agostini et al. (8), Acampa et al. (9, 26), and Giubbini et al. (10), whereas others observed lower values, with stress MBF of 1.11 (interquartile range, IQR: 1.00–1.26) for Nkoulou et al. (7) or 0.67 (IQR, 0.55–0.81) for Zavadovsky et al. (30). This discrepancy between values might result from a lack of standardization in terms of software and corrections (31).

MFR evaluation in SPECT MPI could be considered as an important tool to identify high-risk patients and patients with false negative visual SPECT MPI. Similarly, the SCOTHEART study showed that performing systematic CT coronary angiography in patients referred for CAD screening with suspected angina clearly clarified the diagnostic and resulted in a reduction of fatal and non-fatal coronary events. This helped to identify patients with ongoing CAD even if they were free from ischemia (32). Indeed in those very high-risk patients, pharmacological interventions targeting thrombotic risk and lipid risk were shown to reduce cardiovascular events (13, 33). Aspirin or other antiplatelet drugs are protective in most types of patients who are at an increased risk of atherothrombotic events, including patients with acute myocardial infarction or ischemic stroke, angina (unstable or stable), previous myocardial infarction, stroke or cerebral ischemia, peripheral arterial disease, or atrial fibrillation (34). Its use in primary prevention is more controversial. While in Europe aspirin is not recommended for primary prevention (35), it might be considered in the US (36). Lipid-lowering agents, such as statins, also play a major role in CV prevention. The benefit of lipid-lowering therapy depends on the initial levels of risk; it appears to be greater in patients with a higher risk. There are no differences in the relative reduction between men and women and between younger and older age or between those with and without diabetes (35). Thus, by helping to identify those high-risk patients and improving their prevention strategies, MFR could be a useful tool.

CCS appears to be an independent predictor of CAD (37). It has shown a very high negative predictive value since an Agatston score of 0 has a negative predictive value of nearly 100% for ruling out significant coronary stenosis (38). CCS score and SPECT have already been demonstrated as independent predictors of MACE in patients with suspected coronary artery disease (39). In our study, MFR was significantly reduced in patients with CCS score ≥1. Usually, a CCS score ≥300 Agatston units or ≥75 th percentile for age, sex, and ethnicity is considered to indicate increased CV risk. The Euro-CCAD study showed that the CCS score was a more accurate predictor of significant coronary stenosis than conventional risk factors, with a greater accuracy for predicting >50% stenosis (AUC 0.85) (40). The inclusion of CCS in the Multi-Ethnic Study of Atherosclerosis risk score also offered significant improvements in risk prediction (41). In the European guidelines, CCS scoring should be considered in patients with calculated SCORE risks at around the 5 or 10% thresholds (35). The addition of MFR to CCS could help to better classify patients, especially those with lower or moderate clinical risk.

The combination of MFR, CCS, and myocardial scintigraphy had already been studied by Mentjes et al. (42) and Zampella et al. (43) and showed that the addition of MFR and CCS provided better sensitivity (95%) and better negative predictive value (97%) in the detection of coronary stenosis, supporting the suggestion of Schenker et al. (44) that an imaging approach combining functional and quantitative information offered a clear diagnostic benefit compared to conventional approaches solely based on MPI.

Regarding our dynamic SPECT protocol, we injected 250 and 500 MBq for stress and rest acquisition, respectively, such that even if our total injected dose is higher than those used in some studies [185 and 370 MBq, respectively, at stress and rest for Giubbini et al. (10) and 155 and 370 MBq, respectively, at stress and rest for Acampa et al. (9)], it is still significantly lower than those used by Agostini et al. (3 MBq/kg at rest and 9 MBq/kg at stress) or Nkoulou et al. (330 and 990 MBq) (7, 8). Unlike some authors, our exam protocol consisted in a same-day stress/rest protocol that had the disadvantage of being long for the patients but offered the possibility of multiple prone and/or supine “static” acquisitions, improving the MPI visual image quality by making a definite difference/impact on the inferior wall, with often less digestive uptake or parietal attenuation.

Our study has some limitations that need to be acknowledged; first of all, the small number of patients, especially those having ICA, and the absence of follow-up. Only 102 patients had a successful evaluation of CCS: 89% of the patients scanned in the department equipped with Discovery NM670pro hybrid SPECT and 33% of patients explored in the unit with Symbia T2. This higher failure rate was explained by technological issues inherent to the system technology gap. Then, we have not compared our results to MFR calculated in PET, which remains the gold standard, but as mentioned before, several studies have shown a similar quantification of MBF and MFR using dynamic CZT-SPECT MPI with ^99m^Tc-sestamibi compared to PET (7–10). We also chose to compare our SPECT flow results, which remains a functional test, to an anatomical test used as a reference standard (ICA). This choice may have affected the evaluation of the diagnostic performance of the index test because it is known that the correlation between stenosis severity and myocardial ischemia is poor. The choice to perform ICA or not was left at the cardiologist's decision and might have been influenced by the results of the MPI and flow parameters. Regarding our protocol, the pharmacological stress agent was either dipyridamole or regadenoson, but we previously demonstrated that those two agents induced equivalent hyperemia with similar stress MBF and MFR in comparable patients (45). We did not apply attenuation correction because, in our experience like in other studies, MFR was not different whether it was applied or not (10, 19, 46) and also because most of CZT-SPECT cameras are not equipped with CT, so it may not be achievable in routine. We did not apply motion correction, but before analysis, all our data were screened for motion detection. Finally, we did not perform inter- and intra-observer variability and reproducibility evaluation for MBF and MFR measurements.

## Conclusion

In this study, we showed a significant inverse correlation of MFR with SCORE-estimated 10-year CV mortality risk and number of CV risk factors. MFR also showed excellent performances to predict lesions at ICA.

This suggest that MFR evaluation should be added to SPECT in high- and very-high-risk patients. Thus, even without ischemia on MPI, patients with abnormal MFR might be considered as high-risk patients that need to be confirmed by ICA to further benefit from active secondary prevention. Larger studies with clinical follow-up might be useful to confirm our findings.

## Data Availability Statement

The raw data supporting the conclusions of this article will be made available by the authors, without undue reservation.

## Ethics Statement

The studies involving human participants were reviewed and approved by CPP Ouest III, France. The patients/participants provided their written informed consent to participate in this study.

## Author Contributions

MB, MC, and GM contributed to the conception and design of the study. MB and FT analyzed and interpreted the data. MB drafted the manuscript. MR and DA revised it critically for important intellectual content. All authors have contributed to this work and have read and approved the manuscript.

## Conflict of Interest

MB and GM received honoraria and travel grants from General Electric Healthcare (from previous and other works). DA received honoraria and travel grants from Astra Zeneca, MSD, Amgen, Servier, Sanofi, Bayer, BMS, Pfizer, Boehringer, Novartis, and Novo Nordisk (from previous and other works). The remaining authors declare that the research was conducted in the absence of any commercial or financial relationships that could be construed as a potential conflict of interest.
